# The Road to a Realistic 3D Model for Estimating *R*_2_ and *R*_2_* Relaxation Versus Gd‐DTPA Concentration in Whole Blood and Brain Tumor Vasculature

**DOI:** 10.1002/nbm.5308

**Published:** 2024-12-11

**Authors:** Daniëlle van Dorth, Ahmad Alafandi, Sadaf Soloukey, Pieter Kruizinga, Krishnapriya Venugopal, Aurélien Delphin, Dirk H. J. Poot, Thomas Christen, Marion Smits, Jeroen de Bresser, Juan A. Hernandez‐Tamames, Matthias J. P. van Osch

**Affiliations:** ^1^ C. J. Gorter MRI Center, Department of Radiology Leiden University Medical Center Leiden The Netherlands; ^2^ Department of Radiology and Nuclear Medicine Erasmus MC, University Medical Center Rotterdam Rotterdam The Netherlands; ^3^ Department of Neuroscience Erasmus MC Rotterdam The Netherlands; ^4^ Department of Neurosurgery Erasmus MC Rotterdam The Netherlands; ^5^ Inserm, CHU Grenoble Alpes, CNRS, IRMaGe Université Grenoble Alpes Grenoble France; ^6^ Inserm, U1216, Grenoble Institut Neurosciences, GIN Université Grenoble Alpes Grenoble France; ^7^ Medical Delta Delft The Netherlands; ^8^ Brain Tumor Center Erasmus MC Cancer Institute Rotterdam The Netherlands; ^9^ Department of Radiology Leiden University Medical Center Leiden The Netherlands; ^10^ Department of Imaging Physics TU Delft Delft The Netherlands

**Keywords:** arterial input function, dynamic susceptibility contrast MRI (DSC‐MRI), hematocrit dependency, magnetic field strength, oxygen saturation, simulations

## Abstract

Dynamic susceptibility contrast (DSC) MRI is commonly part of brain tumor imaging. For quantitative analysis, measurement of the arterial input function and tissue concentration time curve is required. Usually, a linear relationship between the MR signal changes and contrast agent concentration ([Gd]) is assumed, even though this is a known simplification. The aim of this study was to develop a realistic 3D simulation model as an efficient method to assess the relationship between Δ*R*_2_^(*)^ and [Gd] both in whole blood and brain tissue. We modified an open‐source 3D simulation model to study different red blood cell configurations for assessing whole‐blood Δ*R*_2_^(*)^ versus [Gd]. The results were validated against previously obtained 2D data and in vitro data. Furthermore, hematocrit levels (30%–50%) and field strengths (1.5–3.0–7.0 T) were varied. Subsequently, realistic tumor vascular networks were derived from intraoperative high framerate Doppler ultrasound data to study the influence of vascular structure and orientation with respect to the main magnetic field (1.5–3.0–7.0 T) for the calculation of Δ*R*_2_^(*)^ versus [Gd] in brain tissue. For whole blood, good agreement of the 3D model was found with in vitro and 2D simulation data when red blood cells were aligned with the blood flow. For brain tissue, minor differences were found between the vascular networks. The effect of vessel direction with respect to *B*_0_ was apparent in case of clear directionality of the main vessels. The dependency on field strength agreed with previous reports. In conclusion, we have shown that the relationship between Δ*R*_2_^(*)^ and [Gd] is affected by the organization of red blood cells and orientation of blood vessels with respect to the main magnetic field, as well as the field strength. These findings are important for further optimization of the realistic 3D model that could eventually be used to improve the estimation of hemodynamic parameters from DSC‐MRI.

## Graphical abstract

Quantitative DSC perfusion MRI requires knowledge of the relationship between *R*_2_^(*)^ relaxation and the concentration of contrast agent. This relationship depends on both physiological and MR parameters, as well as the structure and orientation of the vascular network with respect to the main magnetic field. Our realistic 3D model provides an efficient method to investigate this relationship, and by further optimizing the vascular segmentations, the model could eventually be used to improve the estimation of hemodynamic parameters from DSC‐MRI.

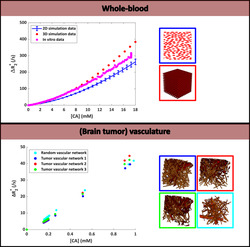

**Abbreviations**
AIF: arterial input function
CBV: cerebral blood volume
DCE: dynamic contrast enhanced
DSC: dynamic susceptibility contrast
RBC: red blood cell

## 1 Introduction

Dynamic susceptibility contrast MRI (DSC‐MRI) is widely used for brain tumor diagnosis and follow‐up. Several studies have highlighted its role in tumor grading, treatment evaluation, and prediction of disease progression [1, 2, 3, 4, 5, 6]. In addition, DSC‐MRI has shown potential to provide relevant information related to the tumor's molecular status as derived from imaging biomarkers [7, 8, 9]. Recently, more studies have been exploring imaging biomarkers that are related to the vascular characteristics in the tumor, which can be derived by the combined use of spin‐echo and gradient‐echo sequences [10]. Combining these two techniques into what is called vessel size imaging or vascular architectural imaging has shown potential for tumor grading and characterization of the tumor's genetic status [11, 12, 13, 14, 15]. While the spin‐echo technique is more specific to the microarchitecture [16], the gradient‐echo sequence remains the preferred method to measure DSC perfusion [17].

An essential step in the quantitative analysis of DSC‐MRI data is to account for the arterial input function (AIF) that represents the delivery of contrast agent into the tissue. Deconvolution of the concentration time curve in the tissue and the AIF yields the residue function, which represents the impulse response function in the tissue after an instantaneous injection of contrast agent. Subsequently, from the residue function, hemodynamic parameters, such as cerebral blood volume (CBV, from the area under the residue function), cerebral blood flow (from the maximal value of the residue function), and mean transit time (from the ratio of CBV over CBF), are estimated. Especially the CBV is a clinically relevant hemodynamic parameter for brain tumor diagnosis and follow‐up.

The AIF and the tissue concentration time curve are generally calculated from the change in *R*_2_* or *R*_2_ relaxation, which is derived from the MR signal changes induced by the contrast agent, under the assumption of linearity between the changes in *R*_2_ or *R*_2_* relaxation and the contrast agent concentration. However, previous in vitro experiments using a gradient‐echo sequence showed evidence of a quadratic relation between *R*_2_* and the contrast agent concentration in human whole blood [18, 19]. In addition, the blood hematocrit level and MR parameters such as sequence type (gradient echo vs. spin echo) and field strength have shown to influence this relation [20, 21, 22, 23, 24]. A strong correlation between hematocrit and *R*_2_* has been demonstrated in vitro [25]. Recently, the influence of the hematocrit and oxygen saturation was studied by using a model that incorporates both the effects of exchange between compartments and the effects of diffusion through the local magnetic field gradients [26]. Blockley et al. [27] concluded that the field strength affected the *R*_1_ and *R*_2_* relaxivities in whole blood for different contrast agents. Besides, the relationship between the *R*_2_* or *R*_2_ relaxation and contrast agent concentration is dependent on whether the measurement is performed in arterial blood or tissue [20].

Although these in vitro experiments provided useful information, they are laborious and therefore not preferred for studying the influence of multiple hemodynamic or MR parameters. Simulations provide an alternative and more efficient approach to study the relation between *R*_2_* or *R*_2_ and contrast concentration. However, most simulation studies used randomly oriented infinite cylinders to model the vasculature, which does not reflect the actual structure and organization of a brain tumor's vascular network. Furthermore, DSC‐MRI simulation studies that incorporate both whole‐blood arterial and microvascular structures are limited.

In our previous work [28], we used an adapted open‐source 2D simulation tool to study the influence of hematocrit level and field strength on the relation between Δ*R*_2_^(*)^ and the contrast agent concentration in whole blood (in this work, the notation Δ*R*_2_^(*)^ referred to both Δ*R*_2_* and Δ*R*_2_, corresponding to the gradient‐echo *R*_2_* relaxation and spin‐echo *R*_2_ relaxation, respectively). The results showed a dependency of this relation on the red blood cell orientation with respect to the main magnetic field. However, the 2D simulation does not capture all aspects of the 3D susceptibility distribution–induced *R*_2_^(*)^ changes, which indicates the need for a more realistic 3D model. Therefore, in this work, we used a 3D extension of the original 2D simulation model to represent a 3D whole‐blood voxel [29]. Additionally, we studied the relation between Δ*R*_2_^(*)^ and the contrast agent concentration not only in whole blood but also in the microvasculature.

The first part of the study focused on the whole‐blood level, for which we validated the 3D simulation setup for the blood signal in a DSC acquisition by comparison with the previously measured in vitro data in whole blood for gradient‐echo sequences [19] and with our 2D simulation results. In the second part of the study, which focused on the tissue level, we incorporated realistic vascular networks of brain tumors in the simulation model, which were derived from intraoperative high framerate ultrafast Doppler ultrasound data [30]. The aim of this study was to establish a realistic 3D model to calculate the AIF and tissue concentration time curve from the *R*_2_* relaxation, which incorporates the characteristics and structure of the vasculature, the blood hematocrit, MR sequence (gradient echo vs. spin echo), orientation of the main magnetic field, and field strength (1.5–3–7 T). The application of the established model on realistic vascular anatomies allows for a more accurate and case‐specific quantitative analysis of DSC‐MRI perfusion data.

## 2 Methods

### 2.1 Simulation Setup

**FIGURE 1:**
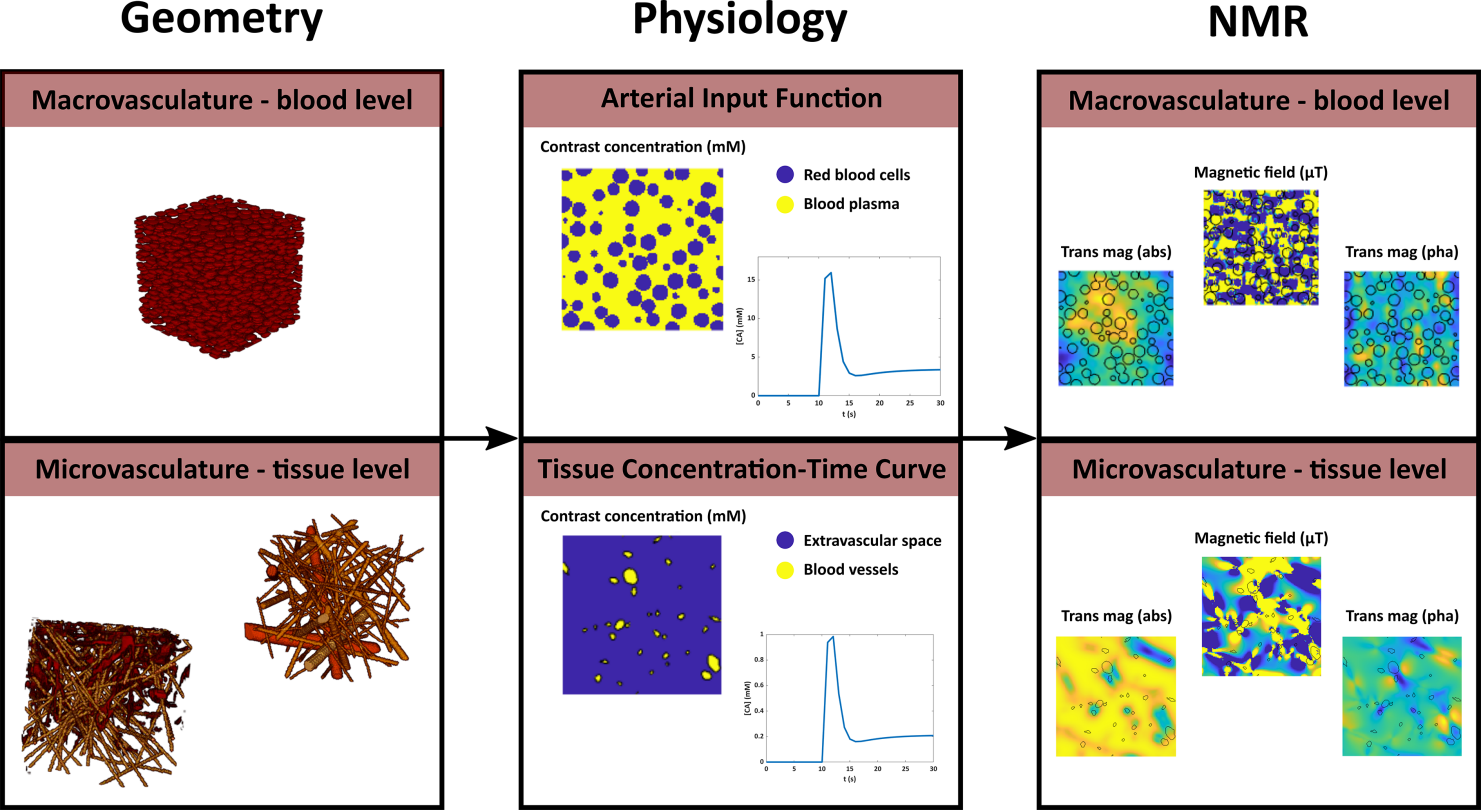
Overview of the simulation model, which consists of three blocks: (1) The geometry block was used to model either the whole‐blood or tissue level, (2) the physiology block simulates the inflow and diffusion of contrast agent, and (3) the NMR block calculates the magnetic field perturbations resulting from the contrast agent effects and the resulting evolution of the magnetization.

The simulations were performed using a privately shared extension to 3D simulations of the MRVox software, which is publicly available on GitHub (https://github.com/nifm‐gin/MrVox2D). The simulation model consists of three blocks, where the first block includes generating or loading a (vascular) geometry. The second block models the inflow, leakage, and distribution of contrast agent in the simulation space. The third block calculates the MR signal changes, where the relaxation effects, susceptibility changes, and the diffusion of water molecules are considered. The susceptibility‐induced magnetic field changes are calculated using a Fourier‐based approach [31, 32]. The diffusion of contrast agent is modeled by a Gaussian diffusion kernel [33, 34]. Some adjustments were made for the small diffusion per time step to avoid too much discretization in sampling the Gaussian, where the diffusion kernel was adjusted such that the intended displacement at a certain point matched that of directly neighboring points. Furthermore, minor changes were made in order to speed up the simulations, for example, the real‐time plots were discarded and calculations of the contrast agent concentrations were only performed for each repetition time (TR = 1 s) instead of every timestep of the simulation (dt = 0.5 ms). Because this study entirely focusses on the *T*_2_ changes, it was assumed that the contrast agent remained in the vasculature. Also, the *r*_1_ relaxivity of the contrast agent was set to zero. Figure 1 provides an overview of the simulation model. Table 1 summarizes the input parameters that were used for our experiments.

**TABLE 1:** Overview of simulation parameters.

Type	Parameter	Value
Lattice	Dimension	128 × 128 × 128 voxels
	Size	256 × 256 × 256 μm^3^
RBCs	Radius	1.25 and 3.5 μm for short and long axes [35]
	Magnetic susceptibility	−0.736 * 10^−6^ ppm cgs (assuming *Y* = 100%) [36]
	*T*_1_	1000 ms^a^
	*T*_2_	Varying with field strength (200/117/50 ms for *B*_0_ of 1.5/3/7 T) [26]
	Proton concentration	0.7
Blood plasma	Magnetic susceptibility	−0.722 * 10^−6^ ppm cgs [36]
	*T*_1_	1000 ms^a^
	*T*_2_	Varying with field strength (1194/500/304 ms for *B*_0_ of 1.5/3/7 T) [26]
	Proton concentration	0.95
Vasculature	Magnetic susceptibility	*χ*_vasculature_ = Hematocrit * (*χ*_oxyHb_ + (1‐SO_2_) * *χ*_Δ(de)oxyHb_) + (1 − Hematocrit) * *χ*_plasma_ with *χ*_oxyHb_ = 4π * (−0.736 * 10^−6^), *χ*_Δ(de)oxyHb_ = 4π * (0.264 * 10^−6^) (difference between fully oxygenated and deoxygenated RBCs) and *χ*_plasma_ = 4π * (−0.722 * 10^−6^) [36]
	*T*_1_	1500 ms^a^
	*T*_2_	70 ms
Contrast agent	*r*_1_ (*T*_1_ relaxivity)	0 mM^−1^ s^−1^ ^a^
	*r*_2_ (*T*_2_ relaxivity)	Varying with field strength (4.6/5.2/4.8 mM^−1^ s^−1^ for *B*_0_ of 1.5/3/7 T) [37, 38]
	Diffusivity	48.5 * 10^−11^ m^2^ s^−1^ (default *D*_free_) [39]
	Permeability	0 s^−1^
	Magnetic susceptibility	4π * 2.55 * 10^−8^ mM^−1^ [19]
MRI	TE	20/40 ms (gradient echo/spin echo)
	TR	1000 ms
	Excitation time	0 s
	FA	90°/90°–180° (gradient echo/spin echo)
	Static magnetic field	Varying (1.5/3/7 T)
Other	Water diffusivity	760 * 10^−12^ m^2^ s^−1^ (default *D*_free_) [39]

^a^ To minimize *T*_1_ effects to reflect complete refreshment of flowing blood.

### 2.2 Whole‐Blood Simulations

The original model generates a geometry consisting of blood vessels and extravascular space. In this part of the study, the geometry that originally corresponded to blood vessels was used to represent the red blood cells, while for the original extravascular space, the model parameters were changed in order to represent the blood plasma. A biconcave shape was applied for the red blood cell geometry. The contrast agent was contained within the blood plasma by simulating an impermeable red blood cell wall. Free diffusion of water was assumed [39]. Initially, the 3D simulation model was validated against the previously obtained in vitro data with a hematocrit of 36% and field strength of 1.5 T [19]. For this purpose, a linearly increasing contrast agent concentration was used with a maximum concentration of 18 mM, corresponding to a double dose of Gd [20, 40]. Subsequently, the changes in *R*_2_* or *R*_2_, collectively referred to as *R*_2_^(*)^, were derived from the following equation:

(1) $${\Delta R}_{2}^{\left(*\right)}\left(t\right)=-\frac{1}{\mathrm{TE}}*\ln\left(\left|\frac{S\left(t\right)}{S_{\text{base}}}\right|\right),$$

where *S*_base_ represents the mean baseline signal magnitude after reaching equilibrium.

The *T*_1_ effects of the contrast agent were ignored in this part of the simulations under the assumption of fresh inflow in a large artery.

Both a random and periodic distributions of red blood cells were applied. The randomly positioned cells were oriented in the same direction by default in the simulation model. In addition, randomly distributed and orientated cells were simulated by the use of the “collision‐driven molecular dynamics” algorithm [41]. This allowed for a more dense packing of cells, thus allowing for a higher maximum hematocrit level, which was previously not possible in the 2D model without causing overlap of cells. The periodic distribution included a zigzag pattern of RBCs to allow for a dense packing of cells and thus also allow for high hematocrit levels. In the next step, the influence of hematocrit level (30%–50%), field strength (1.5–3–7 T), and MR sequence (GRE vs. SE) was examined, where red blood cells with the same orientation, that is, aligned with the flow in the direction of the least resistance [42], were simulated. For the varying field strength, the experiments were repeated with the maximum contrast concentration being scaled by the field strength, that is, the maximum concentration of 18 mM was divided by the ratio of the higher field strength (3 or 7 T) and the field strength of 1.5 T, resulting in a lower maximum concentration at higher field strength.

### 2.3 In Vivo Dataset

Realistic tumor vascular networks of three patients with a glioma were obtained from intraoperative high framerate ultrafast Doppler ultrasound imaging as described by Soloukey et al. [30] The datasets were acquired in accordance with the local IRB regulations and after informed consent was given. Clinical characteristics are provided in Table 2, where it can be seen that the datasets contain tumor vascular networks that were derived from different tumor types. For reference, Datasets 1–3 are corresponding to Patient Numbers 3–5 in the previous work, respectively [30].

**TABLE 2:** Clinical characteristics and tumor diagnosis.

Dataset #	Sex	Age	Diagnosis	Genetic features	Blood volume fraction
1	Male	40	Astrocytoma Grade 2	IDH mutant	2.6%
2	Male	31	Oligodendroglioma Grade 2	IDH mutant, 1p/19q codeleted	3.2%
3	Male	56	Glioblastoma	IDH wild type	4.3%

Postprocessing of these images and segmenting the vascular structures were performed using 3D slicer (https://www.slicer.org). This included removal of background noise by means of a Gaussian low‐pass filter and subsequent thresholding of the images with a minimum threshold of 30 dB to segment the vascular structures. Finally, smoothing segmentation tools were applied to remove connected components that consisted of less than 500 voxels. The resulting vascular segmentations were incorporated into the simulation tool.

### 2.4 Microvascular Simulations

The influence of vascular structure and orientation with respect to the main magnetic field was studied. In this part of the study, a first bolus passage was simulated by using the default gamma variate function included in the original simulation tool, with the following parameters: gamma variate parameter = 7000, *α* = 2.6, *β* = 0.56, dilution time = 5 s. The peak concentration of the AIF was defined as 18 mM, thus the same as for the whole‐blood experiments in the first part of the study. For the vessel orientation experiments, we started by simulating five parallel vessels with the same radius, where the *B*_0_ direction was varied from 0° to 90° (step size of 10°). Next, a single vessel (radius = 20 μm, blood volume fraction = 1%) with an isotropic background of smaller vessels (radius = 2.5 μm, blood volume fraction = 1%) was simulated. These experiments were performed for both gradient echo and spin echo for a field strength of 1.5 T, hematocrit level of 40%, and SO_2_ of 60%. Subsequently, we studied the influence of main vessel orientation with respect to the *B*_0_ field for the datasets described in Table 2. The angle between the main vessels of each dataset and the *B*_0_ field was varied from 0° to 45° to 90°. This was achieved by varying the direction of *B*_0_ with respect to the main vessel direction in each of the datasets, while keeping the datasets unchanged. We focused on the gradient‐echo sequence and thus Δ*R*_2_* for these experiments, because the parameter of interest was the vessel orientation rather than MR sequence in this case. To study the different vascular structures, the three datasets described in Table 2 were compared with each other and with a random vascular network that was generated with the original simulation model. Because the ultrasound technique provided details down to submillimeter resolution, the blood vessels at the smaller capillary level needed to be added to the existing vascular networks to reach a blood volume fraction of 6% for all networks. The additional capillaries were simulated as randomly oriented cylinders with a radius of 2.5 μm. The comparison between the ultrasound derived network and the random vascular networks was performed both with and without the additional capillaries, where for each comparison the blood volume fraction of the random network matched that of the tumor vascular network. Additionally, for one of the tumor vascular networks, the simulations were repeated for varying field strength (1.5–3–7 T) and for both GRE and SE sequences. The experiments for varying field strength were performed with the same maximum concentrations and with the maximum concentration scaled according to the field strength, where the maximum concentration was lower at a higher field strength. For the microvascular experiments, the [CA] concentration corresponds to the concentration in the tissue, thus the averaged concentration over the simulation voxel.

## 3 Results

### 3.1 Whole Blood

**FIGURE 2:**
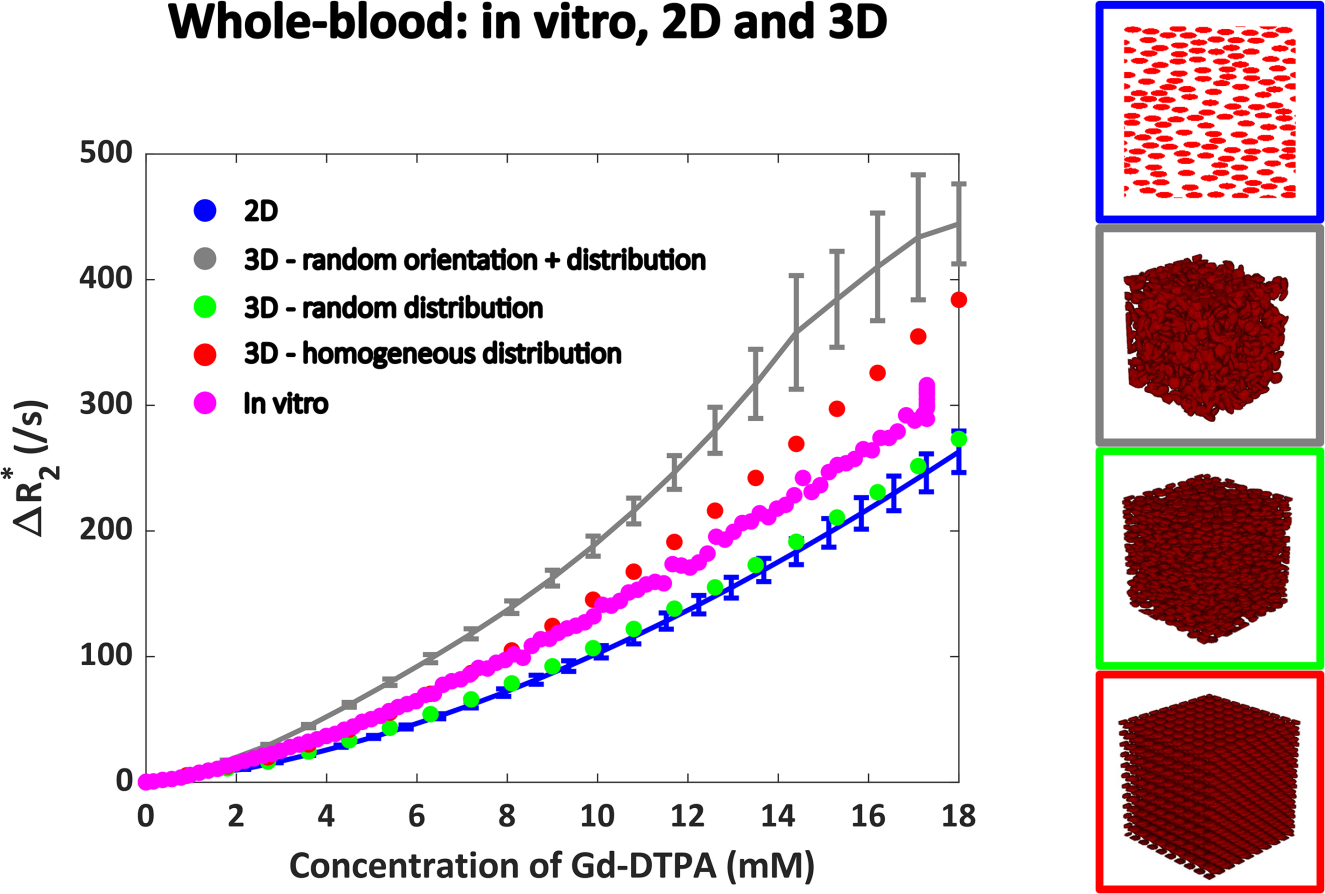
Relationship between Δ*R*_2_* and [Gd] in whole blood, comparing 2D (blue) and 3D (gray, green, and red) simulation models with the in vitro data (pink). The results include the mean and SD calculated over five different RBC configurations, only for the randomly oriented cells in 3D and for the 2D simulations. For random positioning in 3D, both a random (gray) and parallel (green) orientation of RBCs was simulated. Besides, a homogeneous distribution with a zigzag pattern of cells was simulated in 3D (red). The legend images describe the red blood cell configuration, where either a 2D surface plot or a 3D volume plot was created from the binary masks.

Figure 2 shows the relationship between Δ*R*_2_* and [Gd] in whole blood, where the 3D simulations agreed quite well with the in vitro data and previous 2D experiment, though only when red blood cells have the same orientation (red and green datapoints in Figure 2). The results for randomly oriented red blood cells show larger differences with the in vitro data. This is in agreement with previous literature mentioning the alignment of cells with the blood flow in the direction of the least resistance [42].

**FIGURE 3:**
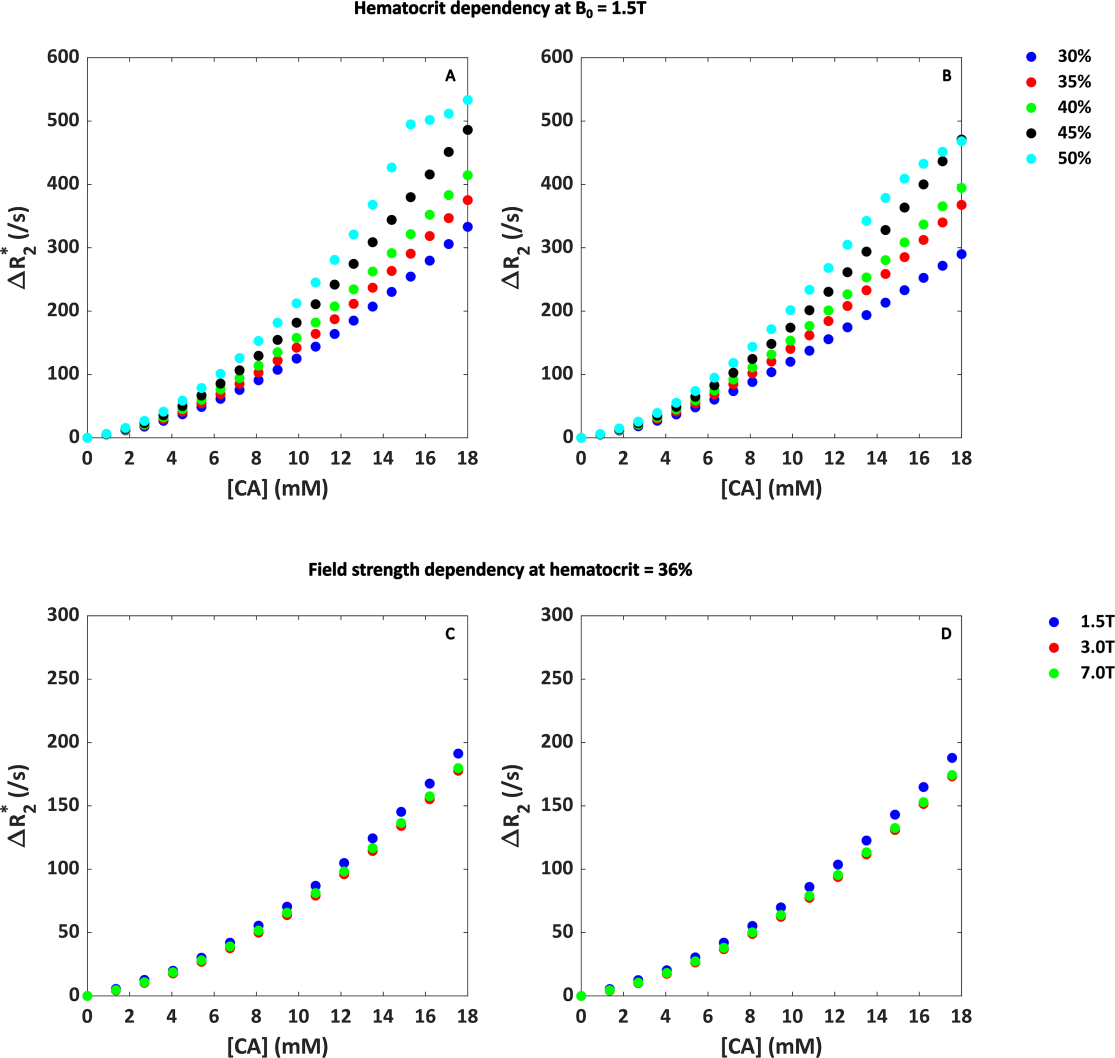
(A) Hematocrit and (B) field strength dependency for the relation between Δ*R*_2_^(*)^ and [Gd] in whole blood. Different colors correspond to different hematocrit levels or field strengths as specified in the legends. In (C) and (D), the maximum contrast concentration was scaled with the field strength, where the maximum concentration was lower at higher field strength. To improve visualization and compare the shape of the curves, the different field strengths were all plotted along the same concentration axis.

The hematocrit and field strength dependency are shown in Figure 3. Figure 3A,B shows a nonlinear relationship across the different hematocrit values at a field strength of 1.5 T, where the relaxivity increases with hematocrit level as expected. For the gradient‐echo sequence at the highest hematocrit level of 50%, the Δ*R*_2_* versus [CA] curve levels off. In Figure 3C,D, the contrast agent concentration was scaled to the field strength by using a higher concentration at lower field strength. To help visualization and allow for comparison of the shape of the curves, the results are plotted against the same concentration axis for each field strength. In these figures, the field strength seems to only minimally affect the relation between Δ*R*_2_* and [Gd], though the scaled curve for 1.5 T displays the highest relaxivity.

### 3.2 Microvasculature

**FIGURE 4:**
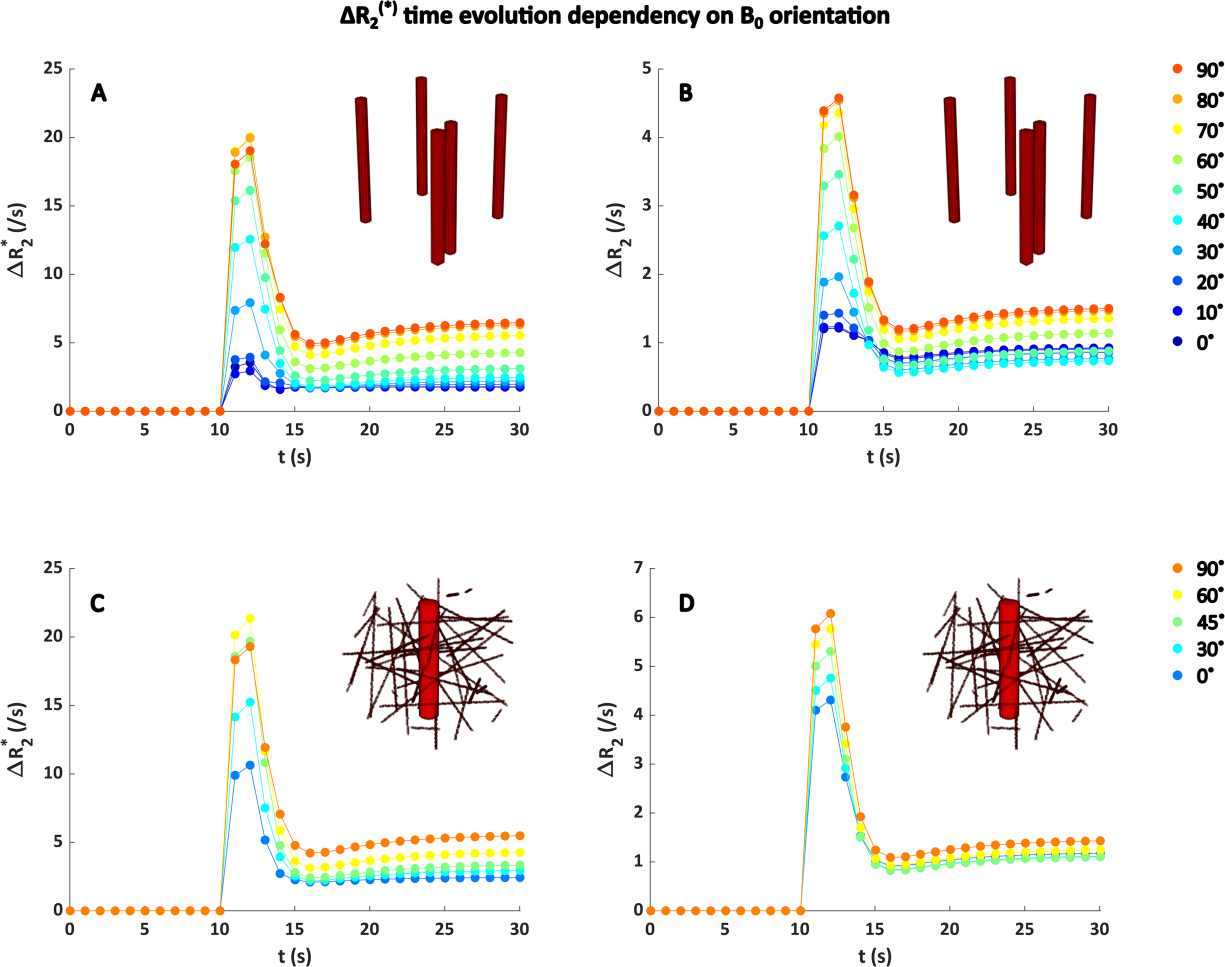
Influence of the orientation to *B*_0_ on gradient‐echo Δ*R*_2_* (A,C) and spin‐echo Δ*R*_2_ (B,D) for a field strength of 1.5 T, hematocrit level of 40%, SO_2_ of 60%, and blood volume fraction of 2%. (A) and (B) show the results for five parallel blood vessels with the same size, whereas (C) and (D) show the results for a single vessel (radius = 20 μm, blood volume fraction = 1%) with an isotropic background consisting of smaller vessels (radius = 2.5 μm, blood volume fraction = 1%). The colors indicate different angles between the blood vessels and the *B*_0_ field. The *x* axis displays the time (s) during which the contrast agent bolus was simulated, whereas the *y* axis shows the corresponding Δ*R*_2_^(*)^ changes. Note the different scales on the *y* axes for gradient echo and spin echo.

The effect of varying the vessel orientation with respect to the *B*_0_ field is shown in Figure 4. For five parallel vessels with the same radius (Figure 4), the effect is clear for both gradient echo and spin echo. As expected from theory, the peak Δ*R*_2_^(*)^ is highest for 90°, because the extravascular field inhomogeneities are most pronounced for a vessel oriented perpendicular to the main magnetic field. Similar results were found for the single vessel with an isotropic background consisting of smaller vessels, although the datapoints for varying angles are closer together.

**FIGURE 5:**
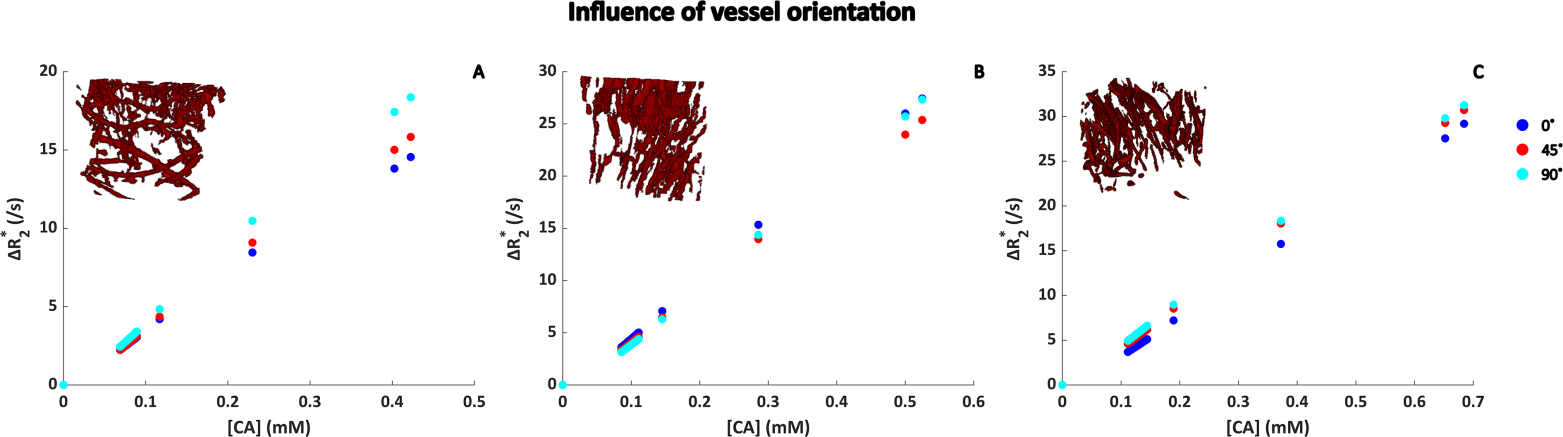
Δ*R*_2_* as function of [CA] for different orientation of the vasculature with respect to the *B*_0_ field in realistic tumor vascular networks. The angle between *B*_0_ and the main vessels was varied between 0°, 45°, and 90°. (A)–(C) correspond to Datasets 1–3 in Table 2, respectively. Note that the maximum CA concentration is different due to the different blood volume fractions for each of the datasets. The CA concentration corresponds to the concentration in the tissue, that is, the average concentration in the simulation voxel.

Next, the dependency on the orientation of the *B*_0_ field with respect to the main blood vessels of the tumor networks was studied for a gradient‐echo sequence (Figure 5). In two of the tumor vascular networks, the highest relaxivity was seen for those cases where the main magnetic field was perpendicular to the main blood vessels, as expected from theory. For Dataset 1, the network with a BVF of 2.6% (Figure 5A), this effect was most pronounced, whereas for Dataset 2 with a BVF of 3.2% (Figure 5B), the datapoints for different angles between *B*_0_ and the main vessels were closer together.

**FIGURE 6:**
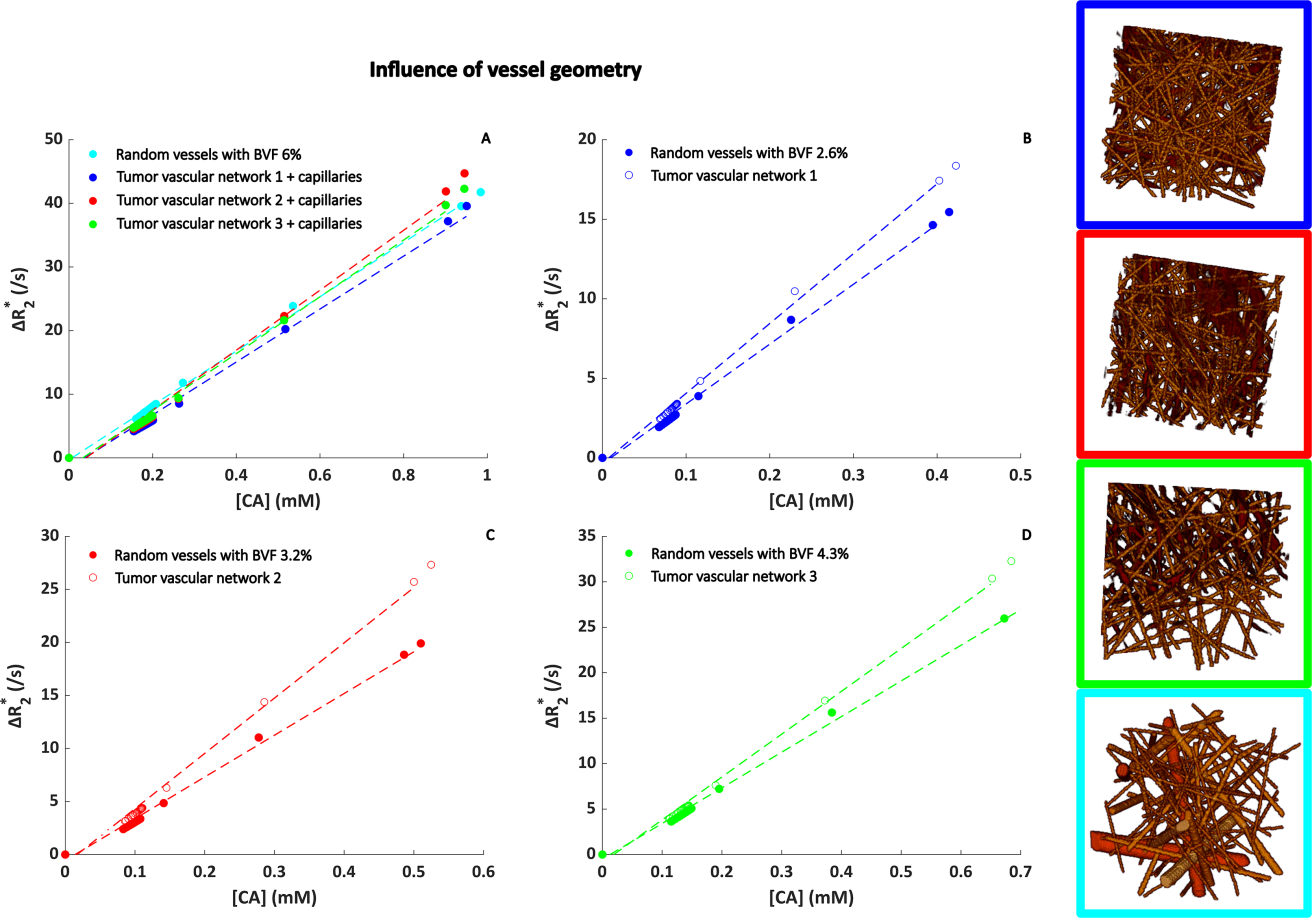
Influence of vessel geometry on the relationship between Δ*R*_2_* and [Gd]. The concentration on the *x* axis is the tissue concentration following a double bolus injection. The segmentations as displayed in the legend images correspond to Datasets 1–3 in Table 2. These were compared to a random vascular network modeled as randomly oriented cylinders. In (A), capillaries are added to the existing tumor vascular networks to make sure the blood volume fraction is 6% for all geometries. In (B)–(D), the original tumor vascular networks (1–3 in Table 2, respectively) are compared to a random network with the same blood volume fraction. Note the different concentration on the *x* axes, which is due to the different blood volume fraction for each of the datasets. The CA concentration corresponds to the concentration in the tissue, that is, the average concentration in the simulation voxel.

Figure 6 shows the relation between Δ*R*_2_* versus [Gd] for the different tumor vascular networks in comparison with a random vascular network. In Figure 6A, the results are shown for the tumor vascular networks with additional capillaries, where it can be seen that the results for the different tumor vascular networks agreed fairly well with the random network with a blood volume fraction of 6%. The obtained relaxivity values from linear fitting were between 0.042 and 0.047 (ms * mM)^−1^ for the tumor vascular networks with additional capillaries compared to 0.043 (ms * mM)^−1^ for the random vascular network. Figure 6B–D shows the results for the tumor vascular networks without the capillaries, compared with random vascular networks with matching blood volume fractions. These results show a more pronounced difference between the random networks and the tumor vascular networks. The fitted relaxivity for Dataset 1 (Figure 6B) was 0.038 versus 0.044 (ms * mM)^−1^ for the random versus tumor vascular network. For Dataset 2 (Figure 6C), this was 0.039 versus 0.052 (ms * mM)^−1^, and for Dataset 3 (Figure 6D), this was 0.039 versus 0.047 (ms * mM)^−1^.

**FIGURE 7:**
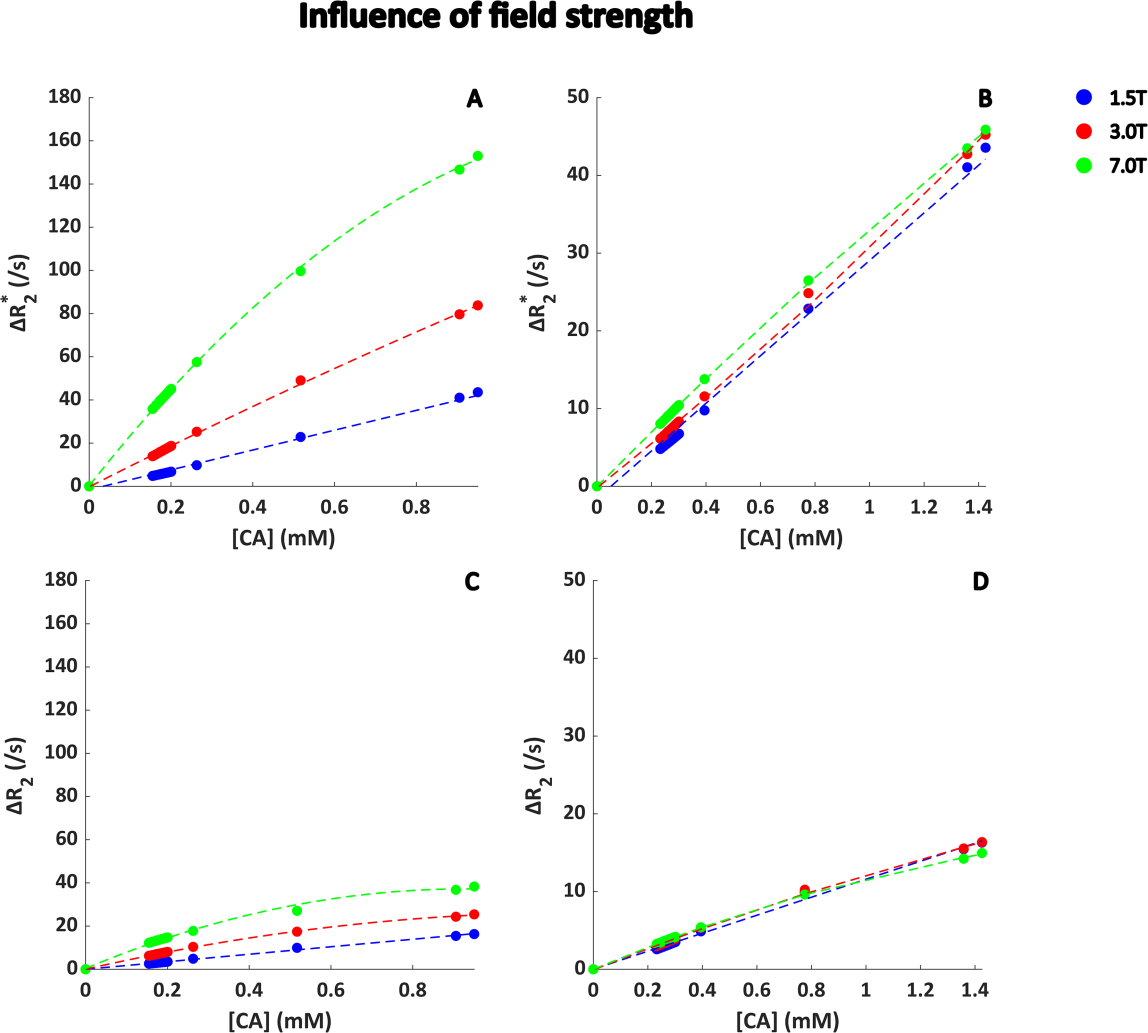
Field strength dependency for the relation between Δ*R*_2_^(*)^ and [Gd] at the tissue level, where a realistic tumor vascular network with additional capillaries was used as input for the simulations. The blood volume fraction was 6%. In (A) and (C), the maximum [CA] was the same for all field strengths, whereas in (B) and (D), the concentration was scaled according to the field strength. For illustration purposes, a first‐ (1.5 T) or second‐order (3.0 and 7 T) polynomial function was fitted and shown as dotted line. The CA concentration corresponds to the concentration in the tissue, that is, the average concentration in the simulation voxel.

In Figure 7, the influence of sequence type (gradient echo vs. spin echo) and field strength (1.5–3–7 T) is summarized. As expected, the relaxivity is lower for the spin‐echo sequence. A higher field strength seems to increase the dephasing effects; however, when the concentration is scaled with the field strength, this effect becomes less apparent. Note that the concentration on the *x* axis is the same for all field strengths also for the scaled curves, which is to help visualization and to allow for direct comparison of the shape of the curves. Interestingly, the linearity of the relation decreases with the field strength, which is mainly visible for the unscaled curves in Figure 7A,C. For the gradient‐echo sequence, the fitted relaxivity values are 0.046, 0.096/0.084, and 0.24/0.25 (ms * mM)^−1^ s^−1^ at 1.5, 3.0, and 7.0 T for the unscaled/scaled concentration, respectively. For spin echo, the fitted relaxivities are 0.017, 0.043/0.042, and 0.079/0.10 (ms * mM)^−1^ at 1.5, 3.0, and 7.0 T for the unscaled/scaled concentration, respectively.

## 4 Discussion

This study was aimed to develop a realistic 3D simulation model for estimating the relationship between Δ*R*_2_^(*)^ and [Gd] in whole blood and brain tumor tissue, which incorporates the dependency on blood hematocrit, vascular geometry, MR sequence type, orientation of the main magnetic field, and field strength.

In whole blood, the relationship between gradient‐echo Δ*R*_2_* and [Gd] agreed well with the in vitro data that was obtained in previous work [19], as well as with the 2D simulation results obtained before [28]. However, this was only the case for red blood cells aligned with the blood flow, where the vessel was assumed to be oriented parallel to the main magnetic field. This agrees to the setup of the in vitro experiment, where the tube with blood was oriented parallel to the main magnetic field. For a random orientation of red blood cells, the overlap of cells could not be entirely avoided, resulting in different shape and size of the objects that are the source of dephasing effects. This overlapping of cells originates from the suboptimal incorporation of the PackLSD algorithm into the MATLAB simulation model. These differences in shape and size depending on the RBC orientation could explain the observed increase in Δ*R*_2_* for a random RBC distribution and orientation compared to the homogeneous distribution. The in vitro data seem to lie in between the random and homogeneous distribution of the red blood cells, which might indicate some random aspect in the red blood cell distribution, though not in their orientation. This agrees with previous literature [42], which states that the red blood cells are oriented along the blood flow in a blood vessel where the flow is high, which holds true for a larger artery such as the internal carotid artery and in the in vitro setup. Therefore, our model assumption for the geometry of the red blood cells seems valid. The relaxivity increased with hematocrit as expected, and, except for a hematocrit level of 50%, a quadratic dependency was found. For the gradient‐echo sequence at the highest hematocrit level of 50%, the Δ*R*_2_* versus [CA] curve leveled off, which could be explained by the simulation model running into digitization noise errors. We observed that the average signal at the highest concentration was only 0.04% of the precontrast baseline signal, mainly due to increased phase dispersion in the simulation volume. The increasing relaxivity with hematocrit agrees to the results of Akbudak et al. [18], where a blood phantom was used to study the dependency on hematocrit in whole blood. In general, when comparing the Δ*R*_2_* values for the same concentration and for hematocrit levels of 30% and 40% at 1.5 T, our values are around 10%–20% higher than found previously. This could be explained by the differences between our simulations and the in vitro setup, because the simulation model lacks the effects of real blood flow (e.g., flow profile) on the behavior of the red blood cells. In contrast to our 2D simulation results, we found a quadratic shape of the Δ*R*_2_^(*)^ curves even at higher field strengths, which is in accordance with previous studies [18, 20, 27]. These findings have important implications for clinical practice, because the linear assumption for AIF calculation would introduce errors in the estimation of the hemodynamic parameters. Previously, Calamante et al. [43] showed that the assumption of linearity results in errors in the CBF calculation of more than 50%. Therefore, including the quadratic dependency and accounting for the physiological and MR parameters that are discussed in our study are expected to largely improve the estimation of hemodynamic parameters from the DSC‐MRI data.

At the tissue level, we observed that the ultrasound derived real vascular networks are less strictly oriented than the random cylinders and the different datasets show clear differences in vascular organization and orientation depending on the tumor type. For the Δ*R*_2_* curves, we found some surprising results with respect to the dependency on vessel geometry. Previous work of Hernández‐Torres et al. [44] showed a significant influence of vessel orientation, which was confirmed by us through simulation of five parallel blood vessels with varying orientation with respect to *B*_0_ and when one large vessel was surrounded by randomly oriented capillaries (Figure 4). However, our main results showing the gradient‐echo Δ*R*_2_* versus [Gd] for the different ultrasound networks showed less pronounced effects (Figure 6). This might be explained by the limitations of the imaging and the segmentation of the vasculature, which are not perfect, that is, there are some structures remaining that might not correspond to vessels and the shape of the vessels seems not perfectly reflected in the segmentations.

When studying the influence of the main vessel orientation with respect to the main magnetic field for the different ultrasound datasets (Figure 5), we found the most pronounced differences for Dataset 1, which is the vascular network with a BVF of 2.6%. This network (as highlighted by the blue box in Figure 6) is characterized by a clear parallel directionality of the main blood vessels surrounded by smaller vessels of which quite some have a similar direction. For Dataset 3, which is the network with a BVF of 3.2% (red box in Figure 6), the vasculature seems more tortuous and almost randomly oriented, which could explain the similar relaxivities for the parallel and perpendicular orientation of the vascular network. In comparison to the previous work of Kjølby et al. [20], where they reported relaxivity values in gray matter of 0.044 and 0.087 (ms * mM)^−1^ at 1.5 and 3 T, our results show reasonable agreement for the observed curves and fitted relaxivity values at 1.5 and 3.0 T of 0.046 and 0.096 (ms * mM)^−1^, respectively (Figure 7).

The results of this study show the potential of using a 3D simulation tool for assessing the relationship between Δ*R*_2_* and [Gd] and is an important addition to the existing literature by incorporating realistic tumor vascular networks.

This study also has some limitations, which are related to the simulation model that could be optimized even further. The first limitation considers the *T*_1_ changes that follow due to the presence and leakage of contrast agent, which are not included in the current model. On the whole‐blood level, the *r*_1_ relaxivity of the contrast agent was set to zero, because in a large blood vessel, we can assume complete refreshment of flowing blood and thus neglect *T*_1_ effects. For the tissue level, this assumption does not hold true, but we still discarded the *T*_1_ effects and especially assumed that all contrast agent remained intravascular, in order to focus on Δ*R*_2_*. Although both *T*_1_ and *T*_2_ changes are important processes that should be considered both for clinical practice, studying them independently before looking at the combined processes is required for better understanding. A next limitation is related to the size of the simulation voxel that is used, which for the whole‐blood experiments only represents a small volume within the artery where the AIF would be measured. However, increasing the simulation volume significantly increases the calculation time and memory that is needed for running the simulations, which is undesirable. The current calculation time for a single experiment is around 8 h with a memory usage of 3 GB. Also, this study focuses entirely on the amplitude of the MRI signal, whereas a phase‐based AIF is known to provide more accurate results and is less affected by the hematocrit level. Contrast agent–induced phase changes depend more on the large‐scale shape and orientation of vessels, which can accurately be modeled or described by analytical formulas when assuming infinite cylindrical shapes [45]. Bridging the small‐ and large‐scale models could in principle be performed by similar simulations as we applied in this study but would be too computational expensive in practice. Moreover, phase‐based AIF measurements have never really been adopted in clinical examinations. Another limitation is related to the flattening off for the Δ*R*_2_* curves at higher concentrations for random distribution and orientation of red blood cells (Figure 2), also seen at higher hematocrit levels for the whole‐blood experiments (Figure 3) and field strength for the microvascular experiments (Figure 7). This behavior seems to originate from discretization noise of the simulation model, because a higher resolution seems to avoid this behavior at high concentrations (see Figure S1).

For the tissue level simulations, the oxygenation was kept the same throughout the vascular networks, although this varies depending on vessel type; thus, it influences the relaxivity in the simulation voxel. Furthermore, oxygenation is also influenced by tumor type and could even vary within the tumor region. However, as the oxygen distribution is independent of the contrast concentration and can be assumed constant in time, including this in our model would only cause a general shift in the data and does not substantially change the Δ*R*_2_^(*)^ values. Another limitation is that the ultrasound data lacks capillary information, because the probe was not sensitive enough to detect the smallest parts of the vascular network. In this study, we added capillaries to the existing vascular networks to attempt making the model more realistic, although this means that any directionality of the capillaries is not accounted for. The structure and directionality of the capillary bed could potentially affect the relationship between Δ*R*_2_* and [Gd], as indicated by the strong dependency on vascular orientation with respect to the main magnetic field in Figures 4 and 5. Future work should therefore focus on optimizing the vascular network segmentations, where the capillary structure and organization should better reflect the realistic scenario. A proof‐of‐concept study already developed a framework for registered ultrasound and fMRI data, which could add information about the location of the vascular structures [46].

## 5 Conclusions

This study shows the development towards a realistic 3D simulation model for calculating the relation between the *R*_2_^(*)^ relaxation and [Gd] in whole blood and brain tumor tissue by studying the influence of vascular structure, orientation of the vascular bed with respect to the *B*_0_ field, field strength, and sequence type. At the tissue level, incorporation of realistic vascular networks could allow for a more patient‐specific and accurate determination of the relationship between Δ*R*_2_^(*)^ and [Gd]. The segmented vascular structures could be improved in further research to better reflect the capillary information of a realistic vascular network. Future work will also include acquiring preoperative MRI data in the same patients and register the ultrasound derived vascular structures to the MRI, thereby providing information about the location of the vascular structures within the tumor. To conclude, the relaxation seems to be affected by the orientation and structure of the tumor's vascular network, which could explain some of the blood volume changes that are observed in clinical practice.

## Supporting information


**Figure S1** Red blood cell simulation with increased resolution of 1 × 1 × 1 μm^3^ compared to the resolution of 2 × 2 × 2 μm^3^ that was used throughout the manuscript. The blue and green curves represent the case where red blood cells are randomly oriented and distributed, whereas the pink and red curves correspond to randomly positioned and parallel oriented cells. Increasing the resolution avoids the leveling off for the case with randomly oriented and positioned cells (blue vs. green curve). The random distribution (pink vs. red) is only slightly affected by the increase in resolution.

## Data Availability

The data that support the findings of this study are available from the corresponding author upon reasonable request.
