# Supplementary material for: The Road to a Realistic 3D Model for Estimating R 2 and R 2* Relaxation Versus Gd‐DTPA Concentration in Whole Blood and Brain Tumor Vasculature
Source: NMR Biomed. 2024 Dec 11;38(1):e5308. doi: 10.1002/nbm.5308 (PMC11635185; doi:10.1002/nbm.5308)
Supplement: Supplementary file 1 — Figure S1 Red blood cell simulation with increased resolution of 1 × 1 × 1 μm3 compared to the resolution of 2 × 2 × 2 μm3 that was used throughout the manuscript. The blue and green curves represent the case where red blood cells are randomly oriented and distributed, whereas the pink and red curves correspond to randomly positioned and parallel oriented cells. Increasing the resolution avoids the leveling off for the case with randomly oriented and positioned cells (blue vs. green curve). The random distribution (pink vs. red) is only slightly affected by the increase in resolution. [file NBM-38-e5308-s001.docx]

# Supplementary Materials

Supplementary Figure 1 shows the results for increasing the resolution from 2x2x2 μm^3^ to 1x1x1 μm^3^ voxels for two configurations of red blood cells. For a random distribution and orientation of red blood cells, the dephasing effects are shown to be larger and we hypothesized that at higher concentrations the simulations enter the discretization noise level, which could explain the leveling off behavior of the curves. For this red blood cell configuration, a clear difference can be seen when increasing the resolution. For randomly positioned, but parallel oriented cells, the simulation does not seem to enter the noise levels for neither a smaller or larger resolution. Overall, these findings support our hypothesis of the discretization noise causing the leveling off for some of the curves at high concentrations.


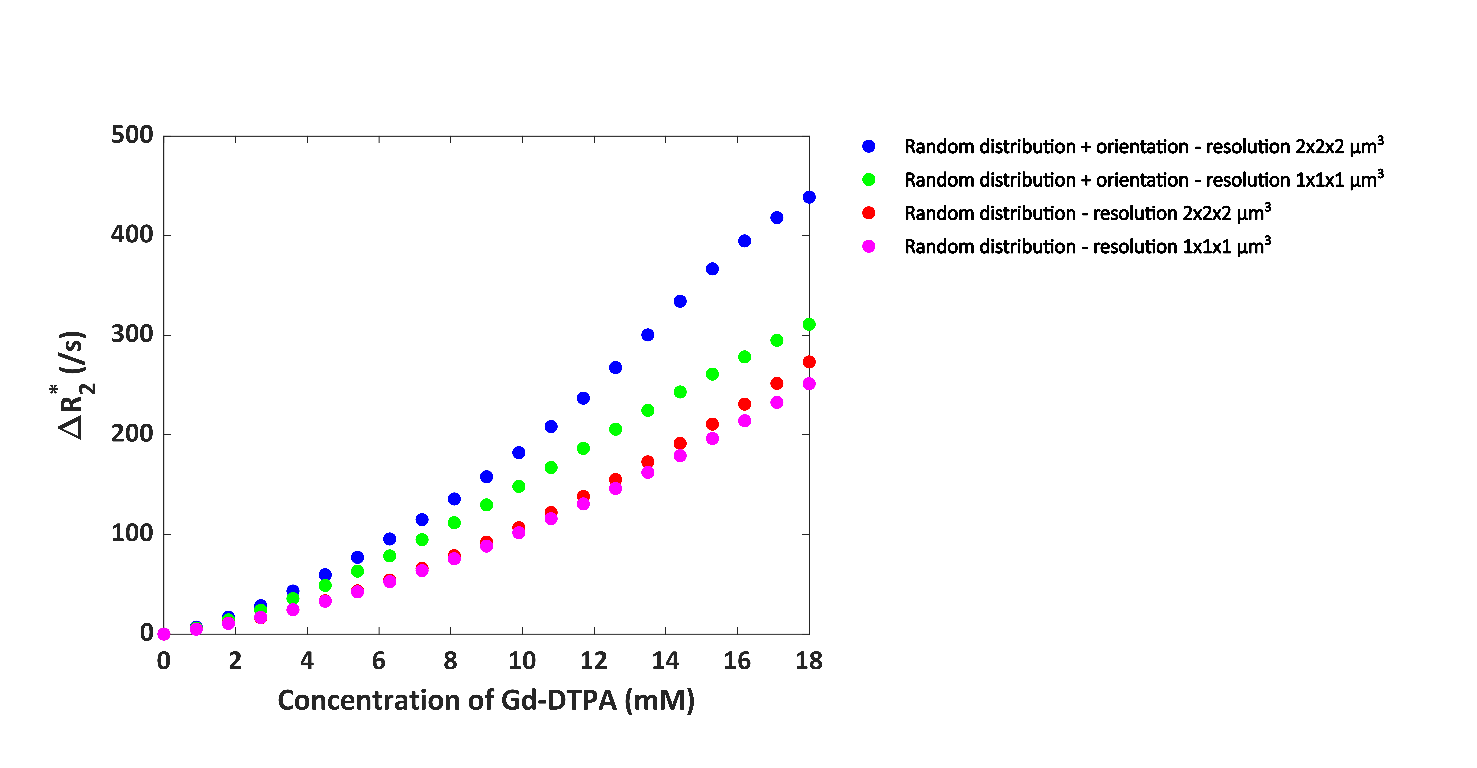


Supplementary Figure 1. Red blood cell simulation with increased resolution of 1x1x1 μm^3^ compared to the resolution of 2x2x2 μm^3^ that was used throughout the manuscript. The blue and green curves represent the case where red blood cells are randomly oriented and distributed, whereas the pink and red curves correspond to randomly positioned and parallel oriented cells. Increasing the resolution avoids the leveling off for the case with randomly oriented and positioned cells (blue versus green curve). The random distribution (pink versus red) is only slightly affected by the increase in resolution.
